# LncRNA LINC00592 mediates the promoter methylation of WIF1 to promote the development of bladder cancer

**DOI:** 10.1515/med-2023-0788

**Published:** 2023-09-29

**Authors:** Tieqiu Wu, Nannan Li, Xinghui Wu, Yongchao Du, Zhiwang Tang

**Affiliations:** Department of Urology, The First Hospital of Changsha, Changsha, Hunan, PR China; Department of Urology, The First Hospital of Changsha, No. 311 Yingpan Road, Kaifu District, Changsha, Hunan, PR China

**Keywords:** bladder cancer, LINC00592, WIF1, promoter, methylation

## Abstract

Epigenetic alteration is a key feature that contributes to the progression of bladder cancer (BC) and long non-coding RNAs serve crucial role in the epigenetic modulation. This study was designed to explore the epigenetic regulation of LINC00592 in BC. LINC00592 expression in BC was examined. Then, LINC00592 was silenced in BC cell followed by cell behavior analyses using CCK-8, transwell, western blot, or flow cytometry. Potential downstream target of LINC00592 was explored using RNA pull-down assay and methylation of WIF1 was determined using methylated-specific PCR. In addition, WIF1 or/and LINC00592 were silenced in BC cells followed by cell behavior analyses to explore the regulation between them. Upregulation of LINC00592 was significantly detected in BC tissues and cells. In BC cells silencing LINC00592 suppressed the proliferation, migration, and epithelial-mesenchymal transitions (EMT), but enhanced apoptosis. Moreover, LINC00592 recruited DNMT1, DNMT3A, and DNMT3B to enhance WIF1 promoter methylation. In addition, WIF1 overexpression suppressed the proliferation, migration, as well as EMT, but enhanced apoptosis. Silencing WIF1 significantly attenuated the role of silencing LINC00592 in suppressing the proliferative, migratory, and EMT ability of BC cells, and increasing the apoptosis. LINC00592 promoted the growth and metastasis of BC via enhancing the promoter methylation of WIF1 and decreasing WIF1 transcription.

## 1 Introduction

Bladder cancer (BC), also called as urological malignancy, is one of the common aggressive cancers worldwide and its incidences has risen steadily in past few decades [1]. The GLOBOCAN data documented that approximately 550,000 BC cases were diagnosed in 2018 [2]. BC is the thirteenth most deadly neoplasm worldwide and contributes to 2.1% of cancer associated deaths, specifically, the mortality rates in male was roughly four times greater than that of female [3]. Despite the development of medical technology, the first-line therapy of metastatic urothelial carcinoma is only based on cisplatin combination, which has unaltered over past few decades. The recurrence and distant metastasis make 5-year survival rate of advanced BC still dismal [4]. Therefore, it is of significance to reveal the mechanism to explore new choices for BC treatment.

Long non-coding RNA (lncRNA) is a type of transcript more than 200 nt in length but without protein coding ability [5]. Accumulating evidence suggested that lncRNA serves critical role in the progression of cancer, via modulating gene transcription, post-transcription, stability, location, and epigenetic modification [6]. LncRNAs also play critical roles in the diagnosis and development of BCs, such as lncRNA CASC11, lncNRA GClnc1, and lncRNA SNHG3 [7–9]. However, the roles of lncRNA in BC remain not fully understood. In 2018, Yuan et al. revealed that LINC00592 is aberrantly expressed in cervical cancer, and is highly associated with the expression of ZNF20, ZNF441, ZNF573, and TMF1, suggesting that LINC00592 might activate cancer progression via modulating transcription or structural integrity [10]. However, the role of LINC00592 in BC is still rarely mentioned.

DNA methylation is a common mammalian cell modification characterized by adding a methyl group at the CpG island using DNA methyltransferases (DNMTs) [11]. Global alterations of DNA methylation have been identified in multiple cancers, and DNA methylation landscape is considered to be a hallmark of cancer development [12]. Chen et al. demonstrated that urine tumor DNA methylation could be used for early diagnosis, minimal, and surveillance of BC [13]. DNA methylation is also considered to be a therapeutic target for BC [14,15]. Recently, Liu et al. have revealed 18 glucose metabolism-related, DNA methylation associated genes, whose associated signatures serve as an independent prognostic factor of BC [16]. Despite these findings, the role of DNA methylation in BC required further elucidation.

Here, the function of LINC00592 in BC was determined in this study. Following this, WIF1 was predicted as a methylation target of LINC00592 and the underlying mechanism was also explored. Based on the investigation, we hope to offer some new evidence for the detection and therapy of BC.

## 2 Methods

### 2.1 Clinical sample collection

A total of 25 pairs of BC and matched non-tumor samples were collected from the First Hospital of Changsha who had undergone operation from June 2021 to September 2022. Once collected, the samples were rapidly stored in liquid nitrogen. All the enrolled patients did not receive neoadjuvant therapy before operation. This investigation was approved by the Ethic Committee of the First Hospital of Changsha and patients have signed the informed consent.

### 2.2 Cell culture and transfection

Normal bladder epithelial cell and human BC cell lines: 5,637, T24, EJ, as well as SW780 were purchased from American Type Culture Collection (VA, USA) and maintained in RPMI-1640 (Invitrogen, Carlsbad, CA, USA) containing 10% fetal bovine serum (FBS, Gibco, Rockville, MD, USA) and 100 U/mL streptomycin–penicillin at 37°C in a humidity cell incubator.

Plasmids of short hairpin negative control (sh-NC), sh-LINC00529, sh-WIF1, overexpression (oe)-NC, and oe-WIF1 were purchased from the Guangzhou RIBOBIO (Guangdong, China). When cell reached 80% confluency, plasmids as candidate were transfected into target cells, respectively, using lipofectamine 2000 (Invitrogen) as suggested. After 48 h of transfection, cells were gathered for transfected efficiency confirmation or the following investigations.

### 2.3 Quantitative real time PCR (qRT-PCR)

Total RNA of tissue or cell samples was extracted using Trizol reagent (Takara, Dalian, China) as suggested. cDNA was synthesized using the Takara reverse transcription kit as per manufacturer’s instruction. Following this, the qRT-PCR was conducted using SYBR Green (Thermo Scientific, MA, USA) on an ABI 7500 instrument (ABI, CA, USA). The qRT-PCR utilized premiers are summarized in Table 1.

**Table 1:** With GAPDH as the internal control, the expression level of certain gene was computed using the 2^−ΔΔCt^

Primer sequences	Forward (5′–3′)	Reverse (5′–3′)
LINC00592	AGCTCATTGGGCTTTTGAGCC	TTACTGTTCATCTTCTGCCTC
WIF1	GGTGCCGAAATGGAGGCTTTTG	GATGCAGAAACCAGGAGTCACAC
GAPDH	TATGATGATATCAAGAGGGTAGT	TGTATCCAAACTCATTGTCATAC

### 2.4 CCK-8 assay

CCK-8 assay (Dojindo, Japan) was carried out to assess the proliferation of cells. First, cells at a density of 5 × 10^3^ per well were seeded in a 96-well plate and cultured for 0, 24, 48, and 72 h. Then, 10 μL of CCK-8 regent was hatched with the cells in each well at 37°C for 1 h. Finally, the optical density of the cells in each well was detected at 450 nm with a microplate reader (Thermo Scientific).

### 2.5 Transwell assay

The 24-well transwell assay (Millpore, Billerica, MA, USA) was used to detect the migration ability. Briefly, cells were collected, calculated, and adjusted in the density of 1 × 10^5^ cells/mL medium without serum. Then, the upper chamber was used to seed cells with 1% FBS and the lower chamber was padded with 500 μL of 10% FBS medium. After 24 h incubation at 37°C, cells in the upper chamber were swabbed using a swab. Then, 4% paraformaldehyde was used to fix the cells on the chamber at room temperature for 15 min followed by 0.5% crystal violet (Sangon, Shanghai, China) staining for 20 min. Finally, the cells were pictured and analyzed under a phase-contrast microscope (Olympus, Tokyo, Japan).

### 2.6 Western blot

Samples were lysed by the radio immunoprecipitation assay (RIPA) lysis buffer (Boster, Wuhan, Hubei, China) containing protease inhibitors at 4°C for 15 min. After 10 min of centrifugation at 4°C, the supernatants were harvested for quantification using the bicinchoninic acid method (Boster). Following this, the protein solution was subjected to 10% SDS-PAGE followed by electro-transferring onto a PVDF membrane. The membrane was blocked with 5% skim milk at indoor temperature for 0.5 h and incubated with the primary anti-WIF1 (1:2,000 dilution; #ab155101; Abcam, Cambridge, UK), MMP-2 (1:2,000 dilution; #ab92536; Abcam), MMP-9 (1:1,000 dilution; #ab76003; Abcam), Vimentin (1:2,000 dilution; #ab137321; Abcam), E-cadherin (1:500 dilution, #ab231303), DNMT1 (1:1,000 dilution; #ab188453; Abcam), DNMT3A (1:500 dilution; #ab2850; Abcam), DNMT3B (1:1,000; #ab2851; Abcam), and GAPDH (1:5,000 dilution; #ab8245; Abcam) at 4°C for 12 h. Subsequently, membrane was hatched with goat anti-rabbit (1:10,000; #ab205718; Abcam) or rabbit anti-mouse (1:5,000; #ab6789; Abcam) secondary antibody for 1 h, and proteins were developed using the enhanced chemiluminescence kit (Millipore) followed by quantification using the Image J Software (NIH, Bethesda, MD, USA).

### 2.7 Flow cytometry

Apoptosis of cells was determined using the Beyotime Annexin-V apoptosis kit (Shanghai, China) as suggested by the manufacturer. After staining, cell samples were subjected to a Attune NxT flow cytometry (Life Technology, MA, USA) followed by a FlowJo V10 CL software (FlowJo LLC, Ashland, OR, USA) analysis.

### 2.8 RNA pull-down

Pierce^TM^ Magnetic RNA-Protein Pull-Down Kit (Thermo) was used for RNA pull-down. Briefly, cell samples were lysed and the magnetic beads were hatched with the 3′-biotin-labeled LINC00592 probes. Following this, the beads were hatched with lysates at 4°C for RNA–RNA binding protein interaction. Then the interacted complexes were eluted, collected, and subjected to western blot assay.

### 2.9 RIP assay

The binding of LINC00592 to DNMTs proteins was examined using a RIP kit (Millipore, MA, USA) according to kit protocol. Briefly, cells were rinsed with phosphate buffer solution and a same volume of RIPA was added to lysate the cells at 4°C for 5 min and centrifuged for 10 min. During centrifugation, 100 μL RIP wash buffer was added to 50 μL of bead, and hatched with 5 μg corresponding antibody (anti-DNMT1, anti-DNMT3A, and anti-DNMT3B) for 30 min at indoor temperature. Then, the magnetic beads-antibody complex was washed, re-suspended in 900 μL RIP wash buffer and incubated with 100 μL cell lysate at 4°C overnight. Subsequently, the magnetic bead–protein complex was harvested and separated by protease K buffer followed by RNA extraction and qRT-PCR.

### 2.10 Methylation-specific PCR (MSP)

DNA sample was isolated using the DNeasy tissue kit (Qiagen, Hilden, Germany). After quantification, 1 μg of DNA was added to 100 μL of water containing 7 μL 3 M NaOH for denaturation at 37°C for 10 min. Then, 550 μL of sodium bisulfite solution provided in the kit was hatched with DNA solution at 50°C water bath for 16 h. Then, DNA samples were purified and amplified with methylated/unmethylated specific primers.

### 2.11 Statistical analyses

Statistical analyses were performed using GraphPad Prism 6.0 (GraphPad Software, CA, USA) and data were presented using mean ± standard deviation. Student’s *t*-test or the one-way analysis of variance followed by Tukey’s *post hoc* test was used for comparison as it was appropriate. Significant difference was considered as *P* < 0.05.

## 3 Results

### 3.1 LINC00592 increased in BC

**Figure 1:**
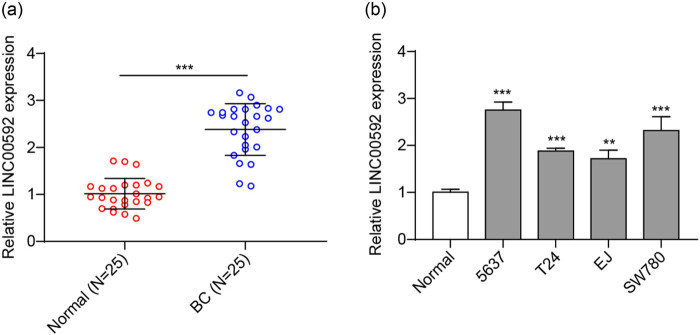
LINC00592 expression is enhanced in BC. (a) LINC00592 expression in BC tissue examined using qRT-PCR. (b) LINC00592 expression in bladder epithelial cell and BC cell lines examined using qRT-PCR. ^**^ *P* < 0.01, ^***^ *P* < 0.001.

First, the expression of LINC00592 was detected by qRT-PCR. It was presented that LINC00592 was significantly enhanced in BC tumor samples in comparison with the adjacent tissues (Figure 1a). Also, LINC00592 expression was markedly upregulated in BC cell lines in comparison with normal bladder epithelial cells (Figure 1b). Specifically, 5,637 cells presented with the highest expression of LINC00592 were thereby chosen for the following investigations. These findings suggested that LINC00592 was significantly upregulated in BC.

### 3.2 Knocking down LINC00592 suppressed the malignant behavior of BC cells

**Figure 2:**
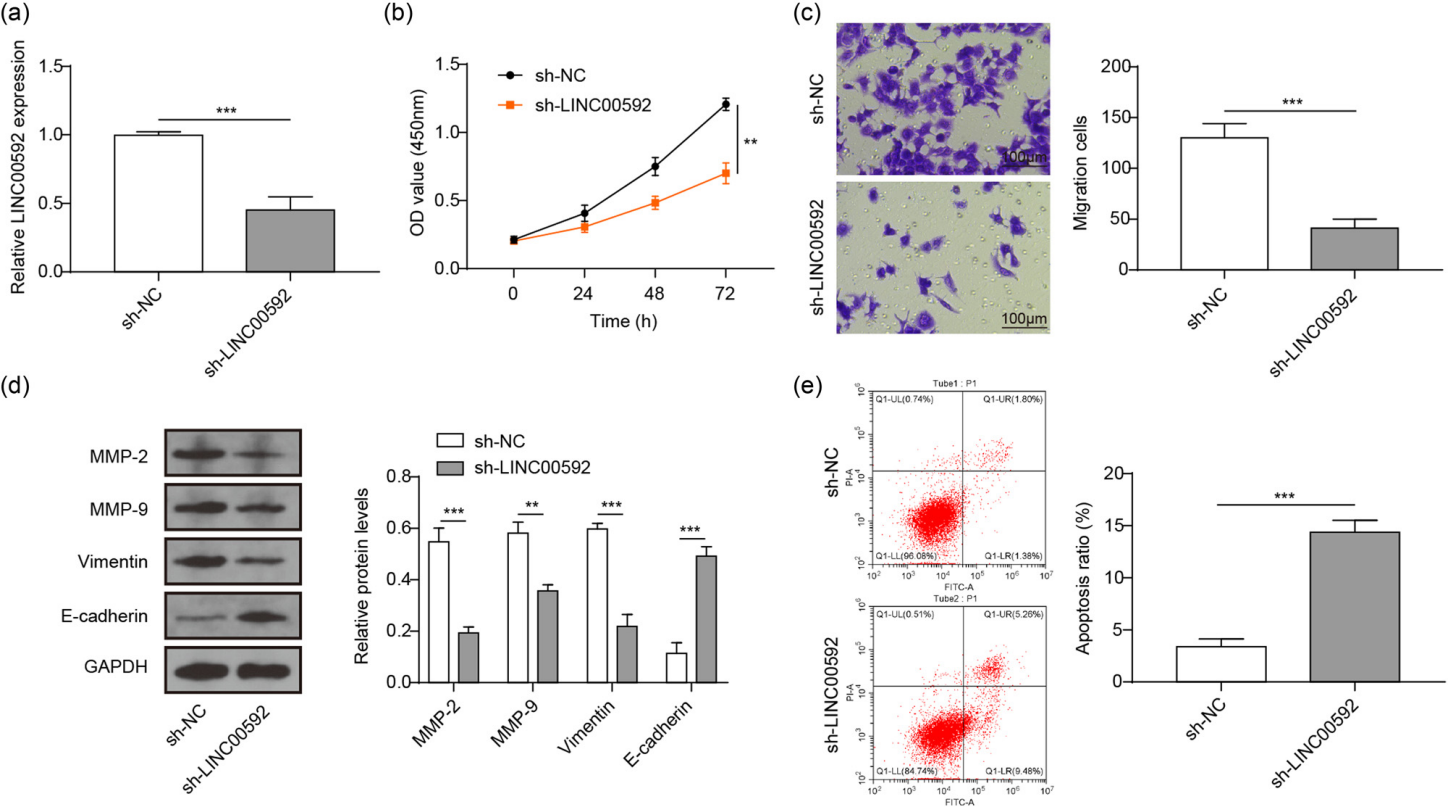
Inhibition of LINC00592 suppressed the proliferation, migration, and EMT of BC cells. (a) Knockdown efficiency of LINC00592 was examined using qRT-PCR. (b)–(e) sh-NC or sh-LINC00592 was transfected into 5,637 cells followed by proliferation, migration, EMT, and apoptosis examinations. (b) Proliferation of 5,637 determined using CCK-8. (c) Migration of 5,637 cells determined using transwell assay. (d) Expression of EMT associated proteins using western blot. (e) Apoptosis of 5,637 cells examined using flow cytometry. ^**^ *P* < 0.01, ^***^ *P* < 0.001.

To reveal the role of LINC00592, LINC00592 was significantly silenced in 5,637 cells (Figure 2a) followed by cell behavior analyses. CCK-8 assay indicated that silencing LINC00592 markedly inhibited the proliferation of 5,637 cells (Figure 2b). Moreover, inhibiting the expression of LINC00592 also significantly decreased the migration of 5,637 cells (Figure 2c). The epithelial-mesenchymal transitions (EMT) associated detection presented that silencing LINC00592 significantly suppressed MMP-2, MMP-9, and Vimentin, but increased E-cadherin (Figure 2d). In addition, suppressing LINC00592 also markedly promoted the apoptosis of 5,637 cells (Figure 2e). These evidence indicated that suppressing LINC00592 might attenuate the proliferation, migration, and EMT of BC.

### 3.3 LINC00592 recruited methylation associated proteins to enhance the methylation of WIF1 promoter

**Figure 3:**
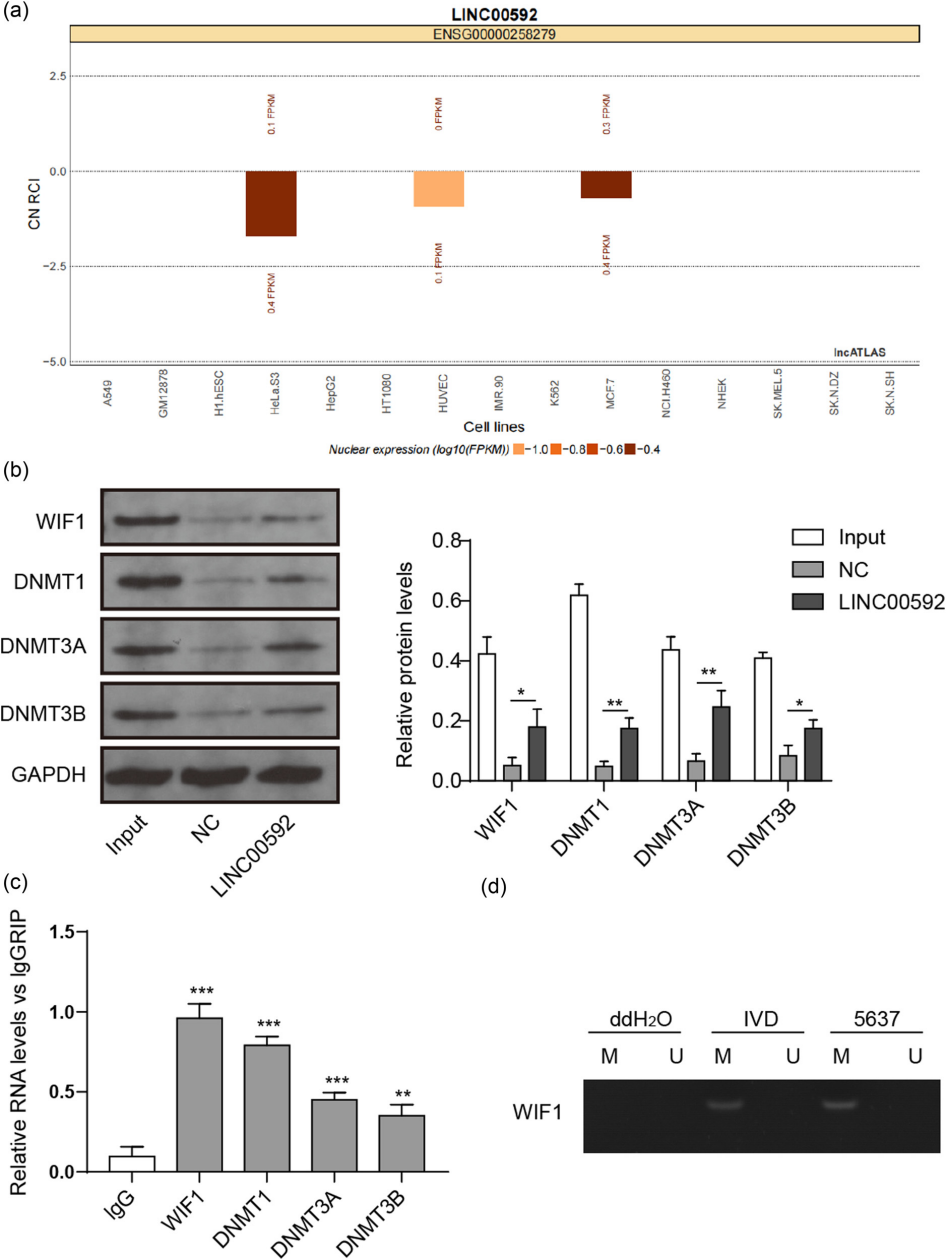
LINC00592 enhances the methylation of WIF1 promoter via recruiting methyltransferases. (a) Location of LINC00592 predicted using the lncATLAS database. (b) LINC00592 interacted or regulated protein determined using RNA pull-down assay. (c) RIP assay determined the interaction between WIF1, DNMT1, DNMT3A, DNMT3B, and LINC00592. (d) Methylation of WIF1 promoter determined using MSP assay. ^*^ *P* < 0.05, ^**^ *P* < 0.01, ^***^ *P* < 0.001.

The lncATLAS analysis revealed that LINC00592 was located in the nucleus (Figure 3a). To further reveal the mechanism of LINC00592, RNA pull-down and RIP assay identified that WIF1, DNMT1, DNMT3A, and DNMT3B were significantly enriched by LINC00592 (Figure 3b and c), suggesting that LINC00592 might involve in methylation regulation. Thus, the methylation of WIF1 was determined and it was found that WIF1 promoter was hypermethylated in 5,637 cells (Figure 3d). These findings indicated that LINC00592 regulated the methylation of WIF1 promoter.

### 3.4 WIF1 overexpression suppressed the malignant behavior of BC cells

**Figure 4:**
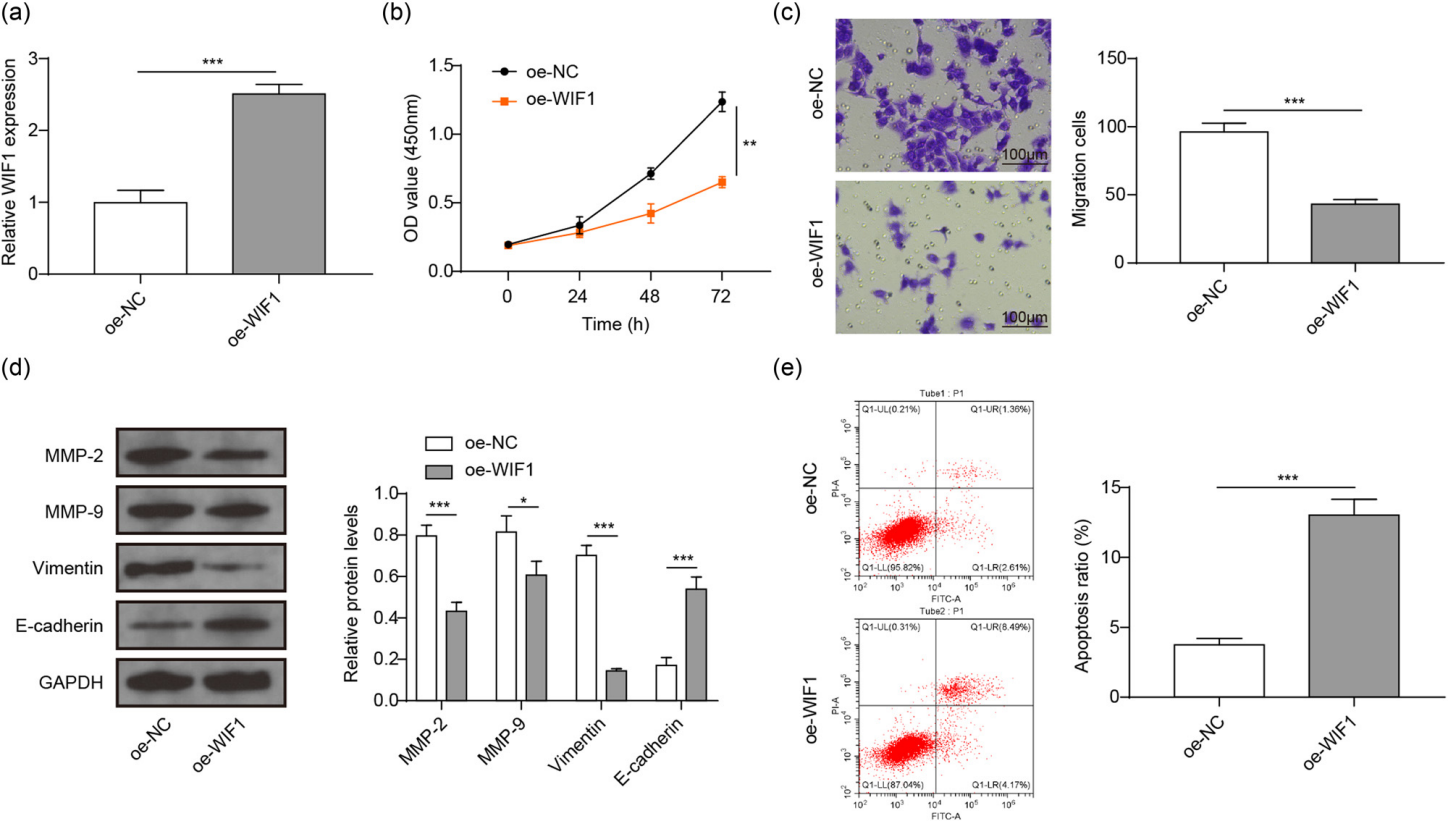
WIF1 inhibits the proliferation, migration, and EMT of BC cells. (a) WIF1 expression was examined using qRT-PCR. (b)–(e) 5,637 cells were transfected with oe-NC or oe-WIF1 followed by proliferation, migration, EMT, and apoptosis analyses. (b) Proliferation of 5,637 cells determined using CCK-8. (c) Migration of 5,637 cells determined using transwell assay. (d) Expression of EMT markers using western blot. (e) Apoptosis of 5,637 cells determined using flow cytometry. ^*^ *P* < 0.05, ^**^ *P* < 0.01, ^***^ *P* < 0.001.

For further exploration, WIF1 was significantly overexpressed in 5,637 cells (Figure 4a) followed by cell behavior analyses. The results indicated that WIF1 upregulation markedly suppressed the proliferation and migration of 5,637 cells (Figure 4b and c). Further detection presented that WIF1 overexpression significantly inhibited the MMP-2, MMP-9, and Vimentin expression, but increased E-cadherin (Figure 4d). Moreover, overexpression of WIF1 obviously promoted the apoptosis of 5,637 cells (Figure 4e). These evidence suggested that WIF1 suppressed the malignant behavior of BC.

### 3.5 LINC00592 promoted the malignant behavior of BC cells via inhibiting WIF1

**Figure 5:**
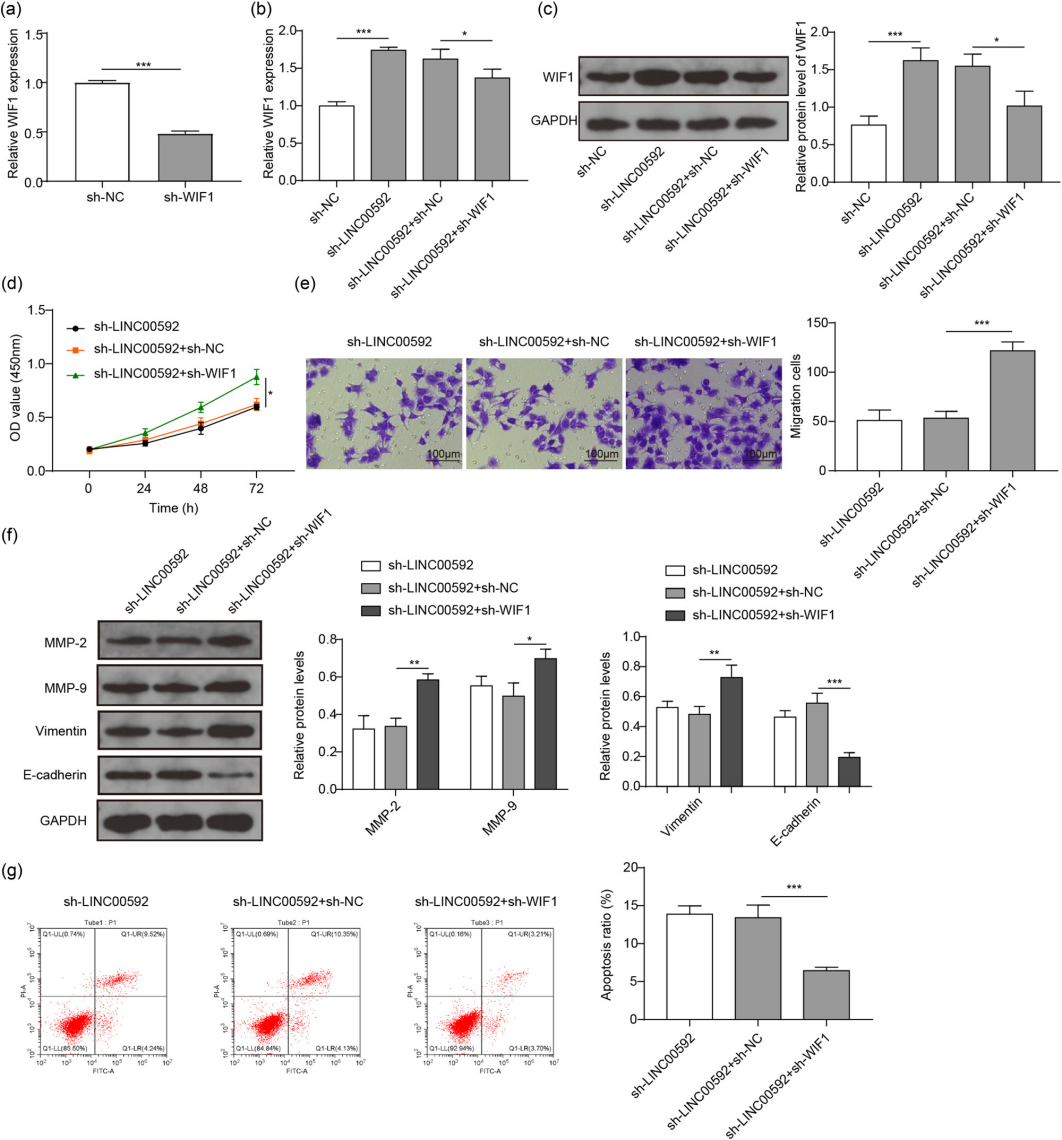
LINC00592 inhibits WIF1 expression to promote the malignant behavior of BC cells. (a)–(g) 5,637 cells were transfected with sh-NC, sh-LINC00592, or/and sh-WIF1 followed by confirmation, proliferation, migration, EMT, and apoptosis analyses. (a) Knockdown efficiency of WIF1 was examined using qRT-PCR. (b) Expression of WIF1 in 5,637 cells was examined using qRT-PCR. (c) WIF1 protein expression in 5,637 cells was detected by western blot. (d) Proliferation of 5,637 cells determined using CCK-8 assay. (e) Migration of 5,637 cells determined using the transwell assay. (f) EMT markers detected using western blot. (g) Apoptosis of 5,637 cells detected by flow cytometry. ^*^ *P* < 0.05, ^**^ *P* < 0.01, ^***^ *P* < 0.001.

To further investigate whether LINC00592 promoted BC cells’ malignant behavior by inhibiting WIF1, rescue experiments were performed. First, qRT-PCR analysis showed that the expression of WIF1 decreased significantly after silencing WIF1 (Figure 5a). Moreover, qRT-PCR analysis showed that silencing LINC00592 significantly enhanced WIF1 expression, while sh-WIF1 attenuated this upregulation (Figure 5b and c). Cell behavior analyses showed that inhibiting WIF1 partially reversed the effect of silencing LINC00592 in mitigating proliferation and migration of 5,637 cells (Figure 5d and e). Western blot analysis showed that sh-LINC00592 significantly inhibited MMP-2, MMP-9, and Vimentin expression, but enhanced E-cadherin expression; while silencing WIF1 partially reversed these changes (Figure 5f). In addition, sh-WIF1 also partially attenuated the effect of sh-LINC00592 on promoting apoptosis of 5,637 cells (Figure 5g). These findings suggested that LINC00592 promoted malignant behavior of BC cells via inhibiting WTF1 expression.

## 4 Discussion

BC is a common neoplasm of the urinary system with its the rapid progression and high rate of recurrence [17]. According to the histological type, BC can be segmented into the muscle-invasive bladder cancer (MIBC) and nonmuscle-invasive bladder cancer, which accounts for ∼70% of newly identified BC, and its 5-year overall survival (OS) can reach ∼90% [18]. However, the 5-year OS for MIBC is only 60–70%, and approximately 10% of them suffered from the distant metastasis with 5-year OS lower than 30% [19]. Epigenetic alteration is a key signature in the progression of cancer and the application of fundamental understanding of the epigenetic dysregulation drives the therapy of BC. In the current study, LINC00592 was identified to involve in the epigenetic regulation of BC via targeting the promoter of WIF1, which provides us a new direction in understanding the proliferation and metastasis of BC.

Accumulated evidence suggested that lncRNAs serve critical functions in the pathogenesis of BC [20]. LncRNA SPRY4-1T1 functions as an miRNA sponge for miR-101-3p to modulate EZH2 expression in BC, thereby contributing to the proliferation and metastasis of BC [21]. LncRNA TUG1 was upregulated in BC and was relevant with the tumor stage, lymphatic metastasis, and prognosis of BC. Mechanism analysis showed that silencing lncRNA TUG1 expression significantly inhibited the hypermethylation of miR-194-5p, thereby attenuating the downstream CCND2 to inhibit the proliferation and to improve cisplatin sensitivity and apoptosis of BC cells [22]. LINC00592 is a newly identified lncRNA located in chr12. Previous studies showed that LINC00592 was involved in immune-related process and iron death associated cell activities [23,24]. In addition, another study documented that LINC00592 was significantly increased in cervical cancer and function analysis presented that LINC00592 might regulate the transcription or structural integrity [10]. However, the exact mechanism was not clearly reported. In this study, RNA pull-down and RIP experiments identified that LINC00592 significantly recruited the WIF1 together with methyltransferases: DNMT1, DNMT3A, and DNMT3B, suggesting that LINC00592 might regulate WIF1 expression via modulating its methylation. Furthermore, MSP and knockdown assay showed that the methylation of WIF1 promoter was markedly increased in BC cells and silencing LINC00592 significantly enhanced WIF1 expression. These evidence suggested that LINC00592 might regulate the progression of BC via targeting the methylation of WIF1 promoter.

WIF1 is a member of the Wnt pathway and has been identified downregulating in several cancers, including prostate, bladder, lung, and breast cancer [25]. Previous study showed that WIF1 promoter was significantly hypermethylated in bladder mucosa samples compared with the adjacent samples, and 5-aza-2′-deoxycytidine treatment markedly decreased the methylation of WIF1 promoter, thereby upregulating WIF1 expression and inhibiting pathogenesis of BC via Wnt/β-catenin [26]. In this study, we have also identified that WIF1 promoter was aberrantly methylated in 5,637 cells and silencing LINC00592 significantly upregulated WIF1 expression. In addition, silencing WIF1 significantly mitigated the effect of shLINC00592 in inhibiting proliferation and migration, indicating that LINC00592 might involve in the WIF1 transcript in BC. In 2019, Jou et al. revealed that WIF1 promoter methylation level was significantly higher in urothelial cells in comparisons with SV-HUC1 cells, and sodium arsenite decreased the WIF1 expression, thereby promoting the migration of BC cells [27]. In this study, we have revealed that overexpression of WIF1 markedly suppressed the growth, metastasis, as well as EMT of BC cells, but accelerated apoptosis. Taken together, LINC00592 enhanced the methylation of WIF1 promoter, thereby decreasing WIF1 expression and its effect on enhancing the growth and migration of BC cells.

## 5 Conclusions

Taken together, this study has documented that LINC00592 is significantly upregulated in BC and WIF1 promoter was markedly hypermethylated in BC. Mechanically, aberrant expression of LINC00592 enhances the promoter methylation of WIF1, thereby downregulating WIF1 expression and decreasing its effect on mitigating the proliferation and metastasis of BC. These findings provided some new evidence on understanding the progression of BC, and targeting the abnormal methylation of WIF1 might be considered as potential biomarker and therapeutic targets of human BC.
